# Expression of bioactive lysophospholipids and processing enzymes in the vitreous from patients with proliferative diabetic retinopathy

**DOI:** 10.1186/1476-511X-13-187

**Published:** 2014-12-11

**Authors:** Ahmed M Abu El-Asrar, Mohd Imtiaz Nawaz, Ghulam Mohammad, Mohammad Mairaj Siddiquei, Kaiser Alam, Ahmed Mousa, Ghislain Opdenakker

**Affiliations:** Department of Ophthalmology, College of Medicine, King Saud University, Riyadh, Saudi Arabia; Rega Institute for Medical Research, Department of Microbiology and Immunology, University of Leuven, Leuven, KU Belgium; Dr. Nasser Al-Rashid Research Chair in Ophthalmology, King Saud University, Riyadh, Saudi Arabia

**Keywords:** Proliferative diabetic retinopathy, Lysophospholipids, Phosphatidic acid, Lysophosphatidic acid, Sphingosine-1-phosphate lyase, Phospholipase A1

## Abstract

**Background:**

The bioactive lysophospholipids phosphatidic acid (PA), lysophosphatidic acid (LPA) and sphingosine-1-phosphate (S1P) have been implicated in mediating cell migration, proliferation and apoptosis, inflammation, angiogenesis and fibrosis. This study was conducted to measure the levels of PA, LPA, LPA-producing enzymes phospholipase A1/A2 (PLA1A/PLA2, respectively) and acylgylycerol kinase (AGK), the S1P receptor S1PR1, the S1P catabolising enzyme S1P lyase (SPL) and 5-lipoxygenase in the vitreous fluid from patients with proliferative diabetic retinopathy (PDR). In addition, we investigated the correlations between the levels of PA and LPA and the levels of the inflammatory and endothelial dysfunction biomarker soluble vascular cell adhesion molecule-1 (sVCAM-1).

**Methods:**

Vitreous samples from 34 PDR and 29 nondiabetic patients were studied by biochemical and enzyme-linked immunosorbent assays and Western blot analysis.

**Results:**

PA, LPA and sVCAM-1 levels in vitreous samples from PDR patients were significantly higher than those in nondiabetic patients. Significant correlations were observed between levels of LPA and levels of PA and sVCAM-1. Western blot analysis revealed a significant increase in the expression of PLA1A, AGK, S1PR1 and SPL in vitreous samples from PDR patients compared to nondiabetic controls, whereas PLA2 and 5-lipoxygenase were not detected.

**Conclusions:**

Our findings suggest that the enzymatic activities of PLA1A and AGK might be responsible for increased synthesis of LPA in PDR and that PLA1A, but not PLA2 is responsible for deacylation of PA to generate LPA. S1PR1 and SPL might regulate inflammatory, angiogenic and fibrogenic responses in PDR.

## Background

Proliferative diabetic retinopathy (PDR) is characterized by inflammation, gradual progressive retinal vasculopathy leading to endothelial cell dysfunction, breakdown of the blood-retinal barrier, ischemia-induced angiogenesis and expansion of extracellular matrix resulting in the outgrowth of fibrovascular membranes at the vitreretinal interface. Formation of fibrovascular tissue often leads to severe visual loss due to vitreous hemorrhage and/or traction retinal detachment. Development of PDR is a complex process where matrix metalloproteinases (MMPs), cytokines, chemokines and growth factors interact with each other to promote inflammation, alterations of the retinal microvasculature, angiogenesis and fibrosis [1–5]. In recent years, bioactive lysophospholipids, such as phosphatidic acid (PA), lysophosphatidic acid (LPA) and sphingosine-1-phosphate (S1P) have been implicated in mediating a variety of biological activities including cell proliferation, migration and apoptosis, inflammation, tumor cell progression and metastasis, angiogenesis, fibrosis and secretion of MMPs, cytokines and chemokines [6–10]. These findings strongly support a role for these molecules in PDR development and progression.

Bioactive lyphophospholipids are synthesized and degraded by a complex set of metabolic pathways. Several routes are proposed for LPA production. LPA can be produced by secretory phospholipase A1 or A2 (PLA1/PLA2, respectively) - catalyzed deacylation of PA [11–13]. PLA2 enzyme also regulates the provision of arachidonic acid to both the cyclooxygenase and lipoxygenase-derived eicosanoids [14]. The other mechanism involves the hydrolysis of the lysophospholipid lysophosphatidylcholine by the lysophospholipase D activity of the ectoenzyme autotaxin. Another proposed pathway for LPA generation is through phosphorylation of monoacylglycerols by a specific lipid kinase, acylglycerol kinase (AGK) [11–13]. In a previous study, we demonstrated elevated levels of LPA and AGK in the vitreous fluid from patients with PDR, whereas autotaxin was downregulated [15]. However, the molecular mechanisms and enzyme pathways involved in LPA production in the diabetic ocular microenvironment are not yet fully understood.

S1P has been well characterized as an agonist of five G-protein coupled receptors, named S1PR1-5. Extensive evidence has accumulated implicating S1P-S1PR1 interaction in regulating cell motility and survival, angiogenesis, vascular maturation and tone, neurogenesis and lymphocyte trafficking [16–18]. In addition, S1PR1 was demonstrated to be a critical component of vascular endothelial growth factor (VEGF)-induced angiogenic response [19]. S1P lyase (SPL) is a stress-induced intracellular enzyme responsible for irreversible degradation of S1P to hexadecenal and phosphoethanolamine. Thus, this enzyme is considered to be a major control point to regulate S1P concentrations in cells [20]. Indeed, constitutive knock-out of SPL in mice leads to a pronounced increase of S1P levels in tissues and serum [21, 22].

The aim of this study was to measure the levels of PA, LPA and the LPA-producing enzymes PLA1, PLA2 and AGK in the vitreous fluid from patients with PDR and to correlate the levels of PA and LPA with the levels of the inflammation and endothelial dysfunction biomarker soluble vascular cell adhesion molecule-1 (sVCAM-1). In addition, we investigated the expression of S1PR1, SPL and 5-lipoxygenase in the vitreous fluid from patients with PDR.

## Results

### Levels of PA, LPA, and sVCAM-1 in vitreous samples

**Figure 1:**
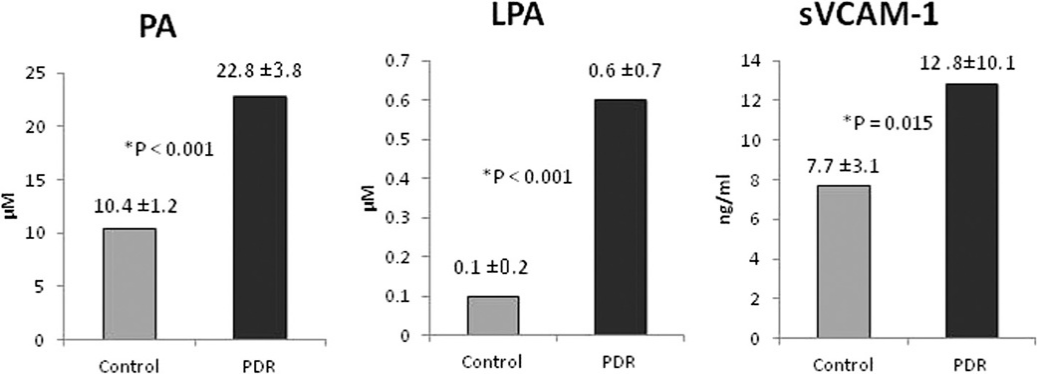
**Comparisons of mean phosphatidic acid (PA), lysophosphatidic acid (LPA) and soluble vascular cell adhesion molecule-1 (sVCAM-1) in vitreous fluid samples from patients with proliferative diabetic retinopathy (PDR) and nondiabetic control patients.** The difference between the two means was statistically significant at 5% level of significance.

With the use of fluorometric assay kit, we detected PA in all analysed vitreous fluid samples from patients with PDR (n = 26) and control patients without diabetes (n = 16). PA mean level in vitreous samples from PDR patients (22.8 ± 3.8 μM) was significantly higher than that in control patients without diabetes (10.4 ± 1.2 μM) (p < 0.0001; Mann–Whitney test) (Figure 1).

With the use of ELISA assay kits, we detected LPA and sVCAM-1 in all analysed vitreous fluid samples from patients with PDR and control patients without diabetes. The mean level of LPA in vitreous samples from PDR patients (n = 26) (0.6 ± 0.7 μM) was significantly higher than that in control patients without diabetes (n = 18) (0.1 ± 0.2 μM) (p < 0.0001; Mann-Whiney test). Similarly, the mean level of sVCAM-1 in vitreous samples from PDR patients (n = 24) (12.8 ± 10.1 ng/ml) was significantly higher than that in control patients without diabetes (n = 25) (7.7 ± 3.1 ng/ml) (p = 0.015; Mann–Whitney test) (Figure 1).

### Correlations

**Figure 2:**
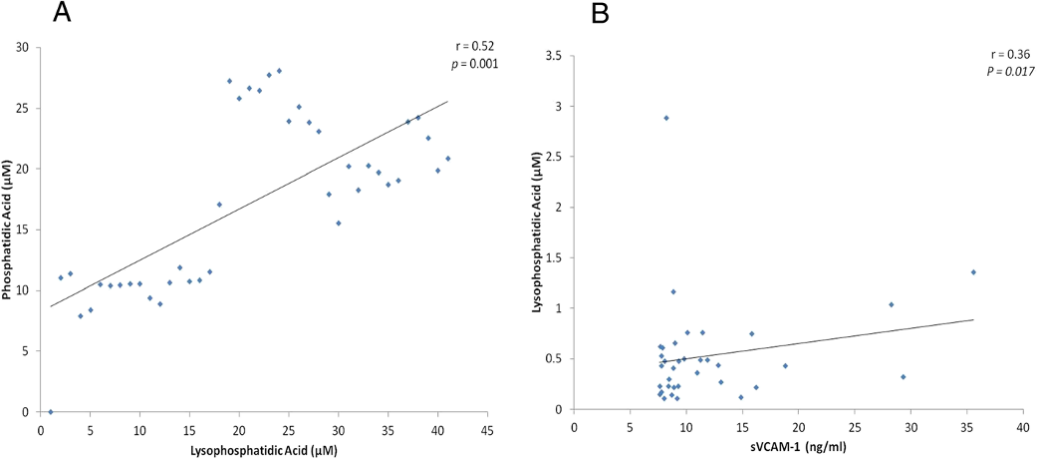
**Significant positive correlations between vitreous fluid levels of lysophosphatidic acid (LPA) and phosphatidic acid (PA) in vitreous samples from 25 proliferative diabetic retinopathy and 15 nondiabetic control patients (A) and soluble vascular cell adhesion molecule-1 (sVCAM-1) in vitreous samples from 22 proliferative diabetic retinopathy and 12 nondiabetic control patients (B).**

There was a significant positive correlation between vitreous fluid levels of PA and LPA (r = 0.52; p = 0.001). In addition, there was a significant positive correlation between vitreous fluid levels of LPA and sVCAM-1 (r = 0.36; p = 0.017) (Figure 2). On the other hand, there was no significant correlation between PA and sVCAM-1.

### Expression levels of PLA1A, PLA2, AGK, S1PR1, SPL, and 5-lipoxygenase in vitreous samples

**Figure 3:**
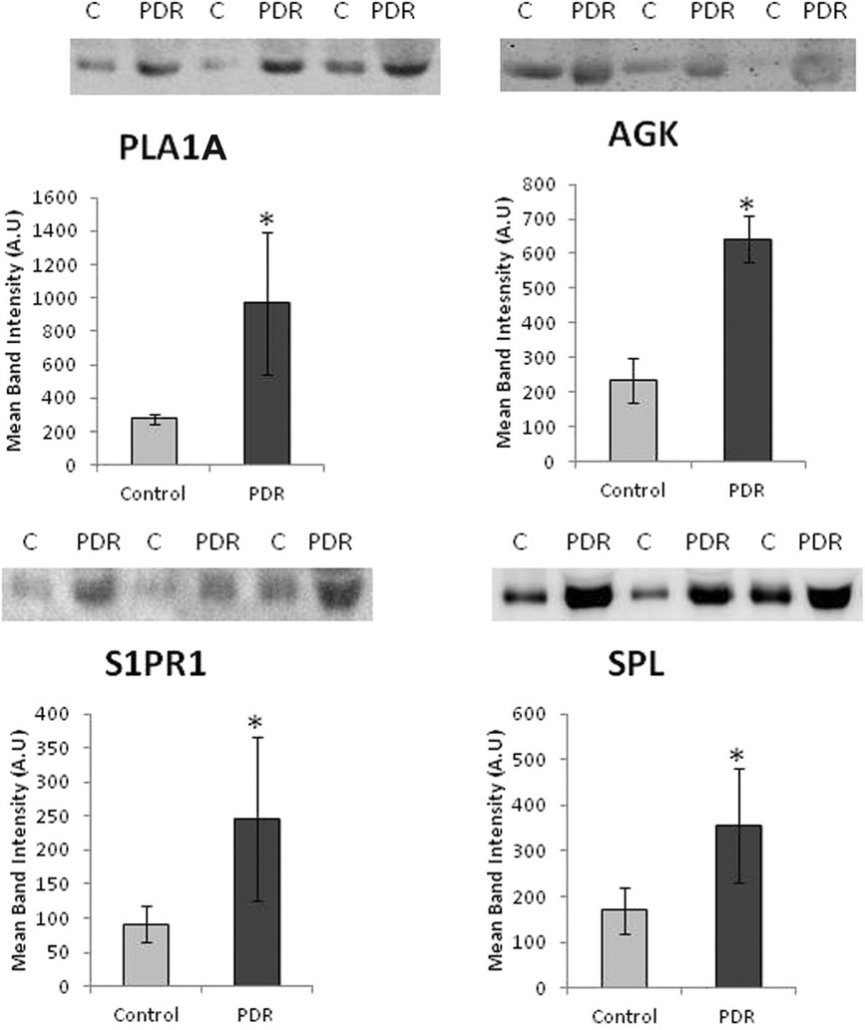
**Comparisons of mean band intensities for phospholipase A1A (PLA1A), acylglycerol kinase (AGK), sphingosine-1-phosphate receptor 1 (S1PR1) and sphingosine-1-phosphate lyase (SPL) in vitreous samples from proliferative diabetic retinopathy (PDR) patients (n = 16) and nondiabetic control patients (n = 16).** A representative set of samples is shown. The difference between the two means was statistically significant at 5% level of significance.

Western blot analysis was used to quantify the expression levels of PLA1A, PLA2, AGK, S1PR1, SPL and 5-lipoxygenase in vitreous fluid samples from patients with PDR (n = 16) and control patients without diabetes (n = 16). We demonstrated that PLA1A, AGK, S1PR1 and SPL were detected in all vitreous fluid samples from patients with PDR and control patients without diabetes (Figure 3).

**Figure 4:**
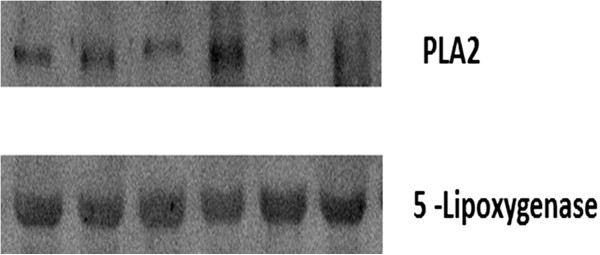
**Western blot analysis of phospholipase A2 (PLA2) and 5-lipoxygenase in serum samples from patients with proliferative diabetic retinopathy.** A representative set of samples is shown. There is expression of PLA2 and 5-lipoxygenase in serum samples.

On the other hand, PLA2 and 5-lipoxygenase were not detected in vitreous fluid samples from patients with PDR and nondiabetic control patients. Western blot analysis was also used to quantify the expression levels of PLA2 and 5-lipoxygenase in serum samples from patients with PDR (n = 12) as a positive control. Western blot analysis revealed expression of PLA2 and 5-lipoxygenase in all serum samples (Figure 4).

Densitometric analysis of the bands demonstrated a significant increase in PLA1A (p = 0.042; Mann–Whitney test), AGK (p < 0.001; Mann–Whitney test), S1PR1 (p < 0.001; Mann–Whitney test), and SPL (p = 0.032; Mann–Whitney test) expressions in vitreous fluid samples from PDR patients compared to control patients without diabetes (Figure 3).

The protein expression of PLA1A in the vitreous samples from PDR patients was increased by about 3.53-fold compared to control patients without diabetes. AGK expression in the vitreous samples from patients with PDR was upregulated by about 2.73-fold as compared to the vitreous samples from control patients without diabetes. The expression of S1PR1 in the vitreous samples from PDR patients was upregulated by about 2.65-fold as compared to the vitreous samples from control patients without diabetes. SPL protein expression in the vitreous samples from PDR patients was increased by about 2.1-fold as compared to control patients without diabetes.

## Discussion

This study shows for the first time increased local levels of PA, PLA1A, S1PR1 and SPL in the vitreous fluid from patients with PDR as compared to nondiabetic control patients, whereas PLA2 and 5-lipoxygenase were undetectable in both diabetic patients and controls. The proinflammatory LPA is a simple potent bioactive phospholipid with many important biological functions, such as mediating cell proliferation, migration and survival, suppression of apoptosis, angiogenesis, fibrosis and MMP-9, cytokine and chemokine secretion [6–8, 23] LPA is a potent activator of several transcription factors and signaling pathways which are known to be involved in the pathophysiology of diabetic retinopathy [24–28]. LPA target genes include those involved in inflammation, angiogenesis and fibrosis [25–27, 29–31] which are the pathological hallmarks of PDR [1–5]. The current data show a significant correlation between the vitreous levels of LPA and that of the inflammatory and endothelial dysfunction biomarker sVCAM-1. Previous studies reported that LPA activates human endothelial cells to upregulate the expression of VCAM-1 [29].

In the present study we report that PA, LPA, PLA1A and AGK were significantly upregulated in the vitreous fluid from patients with PDR, whereas PLA2 was not detected. These results suggest that PLA1A, but not PLA2 might be responsible for deacylation of PA to generate LPA and that PLA1A plays a pivotal role in the development and progression of PDR. In a previous study, we demonstrated that autotaxin was significantly downregulated in the vitreous fluid from patients with PDR and in the retinas of diabetic rats [15]. The results of the present study and our previous report [15] suggest that the enzymatic activities of PLA1A and AGK might be responsible for the increased synthesis and secretion of LPA in PDR. In the present study, 5-lipoxygenase was not detected in the vitreous fluid from patients with PDR suggesting that the PLA2 enzymatic activity is not involved in the progression of PDR.

S1P is a bioactive sphingolipid metabolite and the final common product of complex sphingolipid metabolism. S1P acts through its cognate G protein-coupled receptors to promote cell survival and inhibit apoptosis. S1P-mediated events are also implicated in pathological angiogenesis, inflammation, fibrosis, the response to ischemic injury and cancer progression. In addition, S1P regulates lymphocyte trafficking, promotes DNA synthesis, cell proliferation and cell migration and plays a major role in endothelial integrity. These findings strongly support a role for S1P signaling in promoting tumorigenesis and cancer progression [20]. SPL catalyzes the irreversible degradation of S1P. SPL is, thus, in a strategic position to regulate these same processes by removing the available S1P signaling pools [20]. By reducing S1P pools available for autocrine and paracrine signaling, overexpression of SPL promotes apoptosis under stress conditions including DNA damage [20, 32]. In addition, Huwiler et al. [33] demonstrated that SPL disrupts the biological effects stimulated by extracellular S1P, including aberrant angiogenesis, tumorigenesis, malignant progression and fibrosis.

In the present study, SPL was upregulated in the vitreous from patients with PDR. Recently, several studies reported upregulation of SPL expression in animal models of cardiac ischemia [21] and acute lung injury [22] and in human breast cancer [34]. Bandhuvula et al. [21] demonstrated that cardiac ischemia induced activation of SPL and that inhibition of SPL by either genetic or pharmacologic approaches reduced SPL activity, raised S1P levels, reduced infarct size and increased functional recovery. These findings reveal that SPL is an ischemia-induced enzyme. In a mouse model of acute lung injury, SPL levels are enhanced and S1P levels are decreased. Inhibition of SPL resulted in increased S1P levels, and protected against cytokine release, inflammation and endothelial barrier dysfunction. In addition, it was demonstrated that S1P is a major barrier-protective agent responsible for maintenance of vascular integrity *in vitro* and *in vivo*[22]. These findings suggest that upregulation of SPL in the vitreous fluid from patients with PDR may contribute to diabetes-induced retinal neurodegeneration [35, 36] and vasculopathy. On the other hand, SPL disrupts S1P-induced tumorigenesis and malignant progression, fibrosis and aberrant angiogenesis [33] and loss of SPL expression is significantly associated with aggressive cancers [32, 37]. These findings suggest that the upregulation of SPL in the vitreous fluid from patients with PDR may be a protective antiangiogenic and antifibrogenic eye response to counterbalance the activity of angiogenic and fibrogenic factors in PDR.

Extensive evidence has accumulated implicating S1PR1 in regulating migration of endothelial cells, angiogenesis, survival and lymphocyte trafficking [16–19]. In the present study, we demonstrated that S1PR1 levels were significantly upregulated in the vitreous from patients with PDR. Increased levels of S1PR1 may reflect enhanced tissue S1PR1 expression in the diabetic ocular microenvironment and increased shedding by proteinases as a result of chronic ongoing inflammation. Furthermore, increased levels of S1PR1 in the vitreous fluid from patients with PDR might bind the ligand S1P preventing S1P from reaching the cell surface S1PR1. Our results suggest that elevated levels of S1PR1 potentially negatively regulate S1P signaling and disrupt the biological effects mediated by extracellular S1P.

The vitreous fluid, collected from patients with PDR during pars plana vitrectomy, is an ideal material for analysis of local, intraocular concentrations of selected proteins which take part of this pathology. However, when measuring these factors in the vitreous, some considerations should be kept in mind. Vitreous hemorrhage and breakdown of the blood-retinal barrier can provide an influx of serum proteins into vitreous fluid. However, in a previous study, we demonstrated that there was no correlation between hemoglobin levels, as a measure of the amount of erupted blood, and total protein levels in vitreous fluid from patients with PDR [2]. Although VEGF is abundant in serum, the levels of VEGF in the vitreous fluid did not differ significantly between eyes with nondiabetic massive vitreous hemorrhage and nondiabetic eyes without vitreous hemorrhage. On the other hand, the concentration of VEGF was significantly higher in eyes with PDR than in nondiabetic eyes with massive vitreous hemorrhage or nondiabetic eyes with no vitreous hemorrhage [38]. Furthermore, in a previous study, we reported that brain-derived neurotophic factor (BDNF) was not detected in vitreous samples from patients with PDR and nondiabetic control patients, whereas BDNF was detected in all serum samples from patients with PDR and nondiabetic controls [39]. Similarly, in the present study, we demonstrated that PLA2 and 5-lipoxygenase were not detected in vitreous samples from PDR patients, whereas PLA2 and 5-lipoxygenase were detected in serum samples from patients with PDR. Flow cytometric analysis showed the presence of inflammatory cells in the vitreous fluid from patients with PDR [40, 41]. In addition, endothelial cells, leukocytes and myofibroblasts have been found in fibrovascular epiretinal membranes from patients with PDR [1, 42, 43]. In a previous study, we demonstrated the expression of AGK and the LPA receptor LPA1 by vascular endothelial cells and stromal cells in PDR fibrovascular epiretinal membranes [44]. Taken together, these findings suggest that local cellular production is the relevant source of the studied factors within the ocular microenvironment.

## Conclusions

Our previous study [15], together with the present results, suggest that the enzymatic activities of PLA1A and AGK might be responsible for the increased synthesis of LPA in PDR and that PLA1A, but not PLA2 is responsible for deacylation of PA to generate LPA. Increased expression of S1PR1 and SPL in the vitreous fluid from patients with PDR might regulate the inflammatory, angiogenic and fibrogenic responses in PDR. Our findings suggest that bioactive lysophospholipids could be a novel target in diabetic retinopathy.

## Methods

### Vitreous samples

Undiluted vitreous fluid samples (0.3 – 0.6 ml) were obtained from 34 patients with PDR during pars plana vitrectomy. The indications for vitrectomy were traction retinal detachment, and/or nonclearing vitreous hemorrhage. The diabetic patients were 23 males and 11 females, whose ages ranged from 26 to 86 years with a mean of 50.15 ± 16.7 years. The duration of diabetes ranged from 4 to 30 years with a mean of 18.1 ± 5.9 years. Twenty patients had insulin-dependent diabetes mellitus, and 14 patients had noninsulin-dependent diabetes mellitus. At presentation, the fasting blood glucose was uncontrolled in 22 patients and controlled in 12 patients. Seventeen patients were receiving treatment for hypertension, 9 patients had diabetic nephropathy, and 11 patients had cardiovascular disease. The control group consisted of 29 patients who had undergone vitrectomy for the treatment of rhegmatogenous retinal detachment (RD) with no proliferative vitreoretinopathy. Controls were free from systemic disease and were 20 males and 9 females whose ages ranged from 27 to 66 years with a mean of 49.3 ± 15.5 years. Vitreous samples were collected undiluted by manual suction into a syringe through the aspiratin line of vitrectomy, before opening the infusion line. The samples were centrifuged (5000 rpm for 10 min, 4°C) and the supernatants were aliquoted and frozen at −80°C until assay. The study was conducted according to the tenets of the Declaration of Helsinki. All the patients were candidates for vitrectomy as a surgical procedures. All patients signed a preoperative informed written consent and approved the use of the excised vitreous fluid. The study design and the protocol were approved by the Research Centre and Institutional Review Board of the College of Medicine, King Saud University.

### Enzyme-linked immunosorbent assay kits

Enzyme-linked immunosorbent assay (ELISA) kit for human sVCAM-1 (Quantikine Human soluble Vascular Cell Adhesion Molecules-1, Cat No: DVC00) was purchased from R&D Systems, Minneapolis, MN. LPA (Lysophosphatidic acid, Cat No: K-2800) ELISA was purchased from Echelon Biosciences Inc, Utah, USA. The detection limit for sVCAM-1 was 0.6 ng/mL. The plate readings were done using FLUOstar Omega-Miroplate reader from BMG Labtech, Offenburg, Germany.

### Measurement of human sVCAM-1 and LPA

The quantifications of the level of sVCAM-1 and LPA in the vitreous fluid were determined according to the manufacturer’s instruction. For sVCAM-1 detection, 100 μL of undiluated vitreous samples were added into each ELISA well coated with primary antibody. Following sample incubation, secondary antibody against sVCAM-1, conjugated to horseradish peroxidase was added to each well of the ELISA plate. For LPA, a mix of 100 μL of undiluted vitreous and anti-LPA antibody (sample to antibody at ratio of 4:1) were added in the respective wells. After incubation and washing each well, substrate mix solution of hydrogen peroxide and tetramethyl benzidine (1:1) was added for colour development. The reaction was completed by the addition of 2 N sulfuric acid and optical density (OD) was read at 450 nm in microplate reader. Each assay was performed in duplicate. With the use of a 4-parameter fit logistic curve equation, the actual concentration for each sample was calculated.

### Measurement of human total PA

The quantification of the level of total PA in the vitreous fluid was done according to the manufacturer’s instruction (Cat No: DVC00, Cayman Chemical Company, Ann Arbor, MI). The PA assay kit uses the fluorescence based methods wherein the presence of peroxidase, H_2_O_2_ reacts with ADHP (10-acetyl-3,7-dihydroxyphenoxazine) to give highly fluorescent compound known as resorufin. The total lipids were first extracted from the each vitreous samples and were dried under a gentle stream of nitrogen. The sample was resuspended in 1% triton X-100 and was ready to use for the analysis or storage. After adding 10 μL of each samples in 96 well plate, the reaction was initiated by adding 40 μL of lipase and the plate was incubated at 37°C for 1 hour. The detector mixture was prepared as detailed and 50 μL of which was added to all well and was kept for another 30 minutes at room temperature. The fluorescence was read using an excitation wavelength of 530 nm and emission wavelength of 585 using Gemini XPS fluorescence microplate reader (Molecular Devices, LLC, Sunnyvale, CA). Each assay was performed in duplicate. Using the linear regression curve equation for standard, the concentration for each sample was calculated.

### Western blot analysis for PLA1A, PLA2, AGK, S1PR1, SPL and 5 -lipoxygenase in human vitreous

To determine the expression levels of PLA1A, PLA2, AGK, S1PR1, SPL and 5 -lipoxygenase in the vitreous samples, equal volume of samples were boiled in Laemmli’s sample buffer (1:1, v/v, reducing condition) for 10 min. Equal volume of lysed solution (15 μL) was loaded and separated on 8–10% SDS-polyacrylamide gels (SDS-PAGE) and transferred onto nitrocellulose membranes. After protein transferring, the membrane was blocked (1.5 h, room temperature) with 5% non-fat milk made in Tris-buffered saline containing 0.1%Tween-20 (TBS-T).

For immunodetection of S1PR1, SPL, PLA1A, PLA2, AGK and 5-lipoxygenase, the membrane was incubated overnight at 4°C with rabbit polyclonal anti-S1PR1 (1:300; Cat No:10005228, Cayman Chemical Company), goat polyclonal anti-SPL (1 μg/ml; Cat No: AF5535, R&D Systems), rabbit polyclonal anti-PLA1 (1:1000; Cat No: ab139728, Abcam, UK), rabbit polyclonal anti-PLA2 (1:1000; Cat No: ab23705, Abcam), rabbit polyclonal anti-AGK (1:500; Cat No: ab77266, Abcam) and rabbit polyclonal anti-5-lipoxygenase (1:1000; Cat No: ab39347, Abcam). After overnight incubation with primary antibody, the membrane was washed three times with TBS-T (5 min each). For S1PR1, PLA1A, PLA2, AGK and 5- lipoxygenase the membrane was incubated at room temperature for 1.5 h with anti-rabbit secondary horseradish peroxidase-conjugated antibody (1:2,000, Santa Cruz Biotechnology, Inc., Santa Cruz, CA) and for SPL with anti-goat secondary horseradish peroxidase-conjugated antibody (1:5,000, Santa Cruz Biotechnology, Inc., Santa Cruz, CA). After incubations with secondary antibodies, membranes were washed four times with TBS-T (5 min each) and the immunoreactivity of bands was visualized on a high-performance chemiluminescence machine (G: Box Chemi-XX8 from Syngene, Synoptic Ltd.Cambridge, UK) by using enhanced chemiluminescence plus Luminol (SC-2048, Santa Cruz Biotechnology) and quantified by densitometric analysis using image processing and analysis in GeneTools (Syngene by Synoptic Ltd. Cambridge,UK).

### Statistical analysis

Data are presented as the mean ± standard deviation. The non-parametric Mann–Whitney *U* test was used to compare means from two independent groups. Pearson correlation coefficients were computed to investigate correlations between variables. A p-value less than 0.05 indicated statistical significance. SPSS version 20.0 (IBM Inc., Chicago, IL) was used for the statistical analyses.
